# RETRACTION: Frequent Alterations of LOH11CR2A, PIG8 and CHEK1 Genes at Chromosomal 11q24.1‐24.2 Region in Breast Carcinoma: Clinical and Prognostic Implications

**DOI:** 10.1002/1878-0261.13774

**Published:** 2024-12-03

## Abstract

**RETRACTION:** S. Sinha, R.K. Singh, N. Bhattacharya, N. Mukherjee, S. Ghosh, N. Alam, A. Roy, S. Roychoudhury, and C.K. Panda, “Frequent Alterations of LOH11CR2A, PIG8 and CHEK1 Genes at Chromosomal 11q24.1‐24.2 Region in Breast Carcinoma: Clinical and Prognostic Implications,” *Molecular Oncology* 5, no. 5 (2011): 454–464, https://doi.org/10.1016/j.molonc.2011.06.005.

The above article, published online on 7 July 2011 in Wiley Online Library (wileyonlinelibrary.com), has been retracted by agreement between the journal Editor‐in‐Chief, Kevin M. Ryan; the Federation of European Biochemical Societies (FEBS); and John Wiley & Sons Ltd. Following publication, concerns were raised by a third party that portions of Figure 2 were duplicated, and Figures 5A and 5D were duplicated from an earlier publication by this research group. Internal investigation confirmed the duplications in these figures. The retraction has been agreed because of concerns that the images were manipulated, affecting the interpretation of the data and results presented.

**RETRACTION:** S. Sinha, R.K. Singh, N. Bhattacharya, N. Mukherjee, S. Ghosh, N. Alam, A. Roy, S. Roychoudhury, and C.K. Panda, “Frequent Alterations of LOH11CR2A, PIG8 and CHEK1 Genes at Chromosomal 11q24.1‐24.2 Region in Breast Carcinoma: Clinical and Prognostic Implications,” *Molecular Oncology* 5, no. 5 (2011): 454–464, https://doi.org/10.1016/j.molonc.2011.06.005.

The above article, published online on 7 July 2011 in Wiley Online Library (wileyonlinelibrary.com), has been retracted by agreement between the journal Editor‐in‐Chief, Kevin M. Ryan; the Federation of European Biochemical Societies (FEBS); and John Wiley & Sons Ltd. Following publication, concerns were raised by a third party that portions of Figure 2 were duplicated, and Figures 5A and 5D were duplicated from an earlier publication by this research group. Internal investigation confirmed the duplications in these figures. The retraction has been agreed because of concerns that the images were manipulated, affecting the interpretation of the data and results presented.

